# What characterizes first-time use of eldercare? A SNAC Stockholm Eldercare registry-based study of older people’s health and care needs in Sweden

**DOI:** 10.1186/s12913-026-15500-3

**Published:** 2026-09-02

**Authors:** Pernilla Alencar Siljehag, Åsa von Berens, Bettina Meinow, Carin Lennartsson, Janne Agerholm

**Affiliations:** 1 Stockholm Gerontology Research Center, Stockholm, Sweden; 2 Stockholms Gerontology Research Center, Aging Research Center, Karolinska Institutet, Stockholm University, Stockholm, Sweden

**Keywords:** Long-term care, Home care services, Primary health care, Health care, Older adults, Coordinated care

## Abstract

**Background:**

As populations age, it is becoming more important than ever to understand how older people’s care needs can be met and to ensure that local care providers are coordinated effectively. Describing older adults’ demographic characteristics, functional status, health status, and patterns of healthcare use leading up to their first-time use of eldercare provides valuable knowledge. This can facilitate the mobilization of expertise and methods in eldercare and healthcare and contribute to the joint planning of proactive and more targeted interventions for older adults.

**Methods:**

Data on demographics and functional status of people ≥ 65 years in first-time eldercare (July 2021 to June 2022; *n* = 3649) were obtained from the SNAC Stockholm Eldercare study and combined with registry data on medical diagnoses and use of inpatient and outpatient care six months preceding eldercare entry. Data were summarized using descriptive analyses. The RECORD checklist was used as a reporting guide.

**Results:**

The average age was 80 years. More than half were women and lived alone. One in four people had developed extensive functional limitations. Multimorbidity was common (92%) and almost half had as many as seven or more chronic conditions. Psychiatric comorbidity was prevalent in 15% of cases. Most people received informal care on a weekly or daily basis. Primary care was the predominant form of outpatient care, used by 80% of individuals before entering eldercare. The vast majority (95%) received home care. Every second person entered eldercare immediately following inpatient care. Many individuals in this group had experienced serious injuries, deterioration of existing chronic diseases or temporary infections.

**Conclusions:**

Many older people enter eldercare with extensive and complex care needs and receive support and care from several providers. These findings highlight which needs and care contexts during the period preceding the first use of publicly funded eldercare services are particularly important to consider when developing timely and well-coordinated support. They are useful to guide collaborative strategies, facilitating the use of expertise and methods of both municipal and primary care actors.

**Supplementary Information:**

The online version contains supplementary material available at https://doi.org/10.1186/s12913-026-15500-3.

## Background

With a gradual deterioration of functional capacity in old age, the importance of access to locally provided and well-coordinated healthcare and eldercare services with good continuity increases [1–3]. This is a challenge worldwide with an expected 100% global increase of people aged 60 + years, from 1 billion in 2015 to 2.1 billion in 2050 [4]. This descriptive study provides an up-to-date, population-based profile of older people’s life circumstances, care needs, and use of care and support services when they first access eldercare in Stockholm. Such profiling can guide service planners to match needs to available services, support local care providers in coordinating their activities, and guide the appropriate allocation of welfare resources. This article contributes to that knowledge by offering a comprehensive overview of the situation of older adults in the region.

**Fig. 1:**
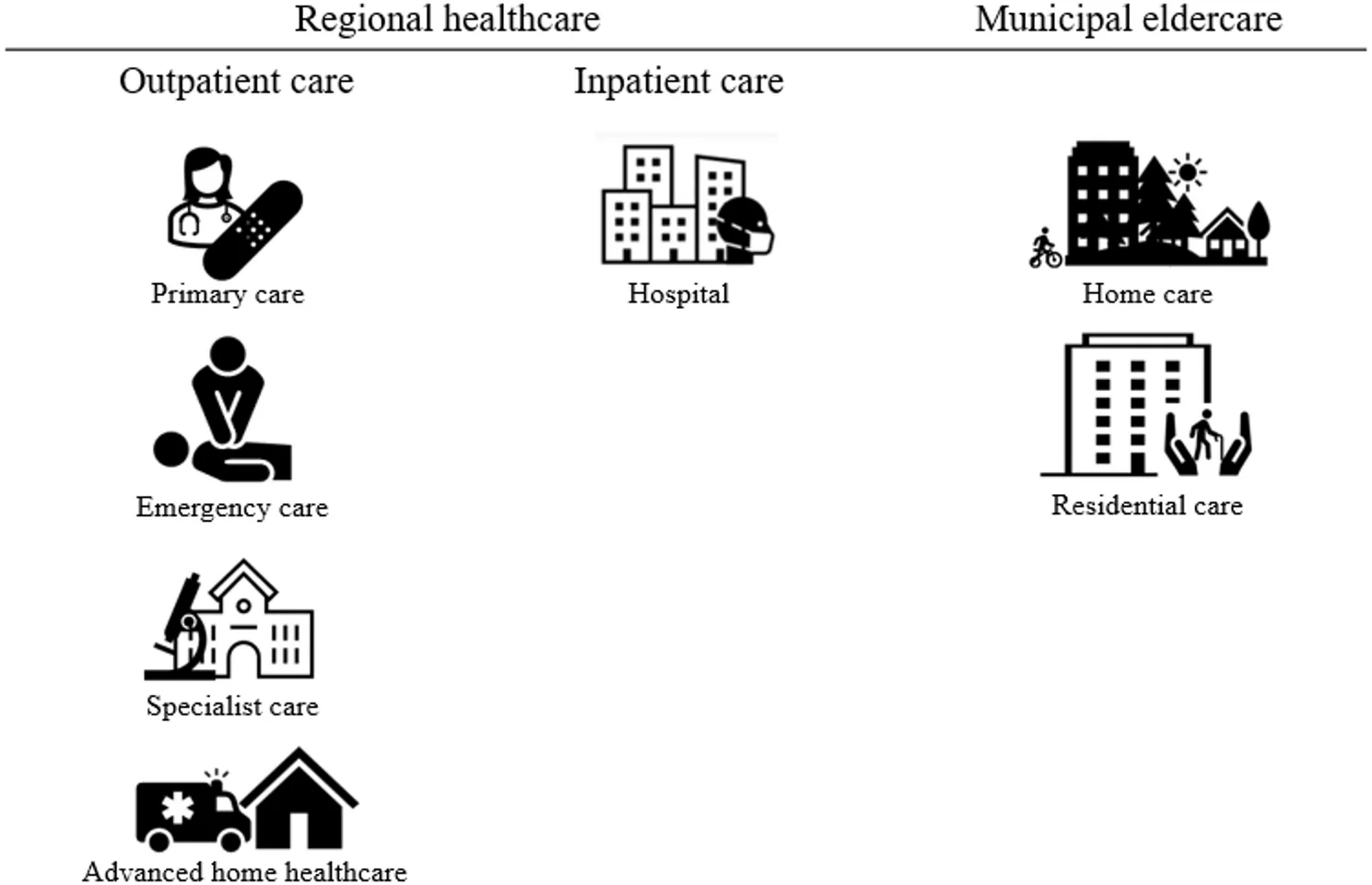
Overview of the Swedish health and eldercare program for older people

In Sweden, the health and eldercare program for people 65 years and older is comprehensive and publicly funded. Healthcare is primarily provided by the regions while eldercare is provided by the municipalities [5] (Fig. 1).

Swedish healthcare is organized at the regional level, meaning that Region Stockholm is responsible for providing outpatient and inpatient care across the 26 municipalities in the county. Fee-based forms of healthcare are substantially subsidized and covered by an out-of-pocket expenditure cap. For some groups, such as children and people older than 85 years, healthcare is free of charge. Primary care, organized in multiprofessional teams, has been assigned the role of coordinating collaborative interventions when both municipal and healthcare services are required in the care and support of an older person [6–8]. Through routine patient contacts, primary care often identifies early signs of an older person’s need for care and support, sometimes before the individual applies for municipal eldercare services.

The nationwide and largely tax-funded eldercare program, which provides social care and is available to all, regardless of socioeconomic status, is also used by the great majority of Swedes at some point during life [9]. Individual costs are income-based and capped at €235 per month [10]. There are no private eldercare systems, such as health insurance-based alternatives, that operate in parallel with the public system to provide personal care. The main forms of eldercare are home care (formal social care provided in ordinary housing) and residential care (institutional placement providing both social care and healthcare). Services are provided in accordance with grant decisions made on the basis of functional need assessments carried out by the municipality, often by social workers [10]. However, even in this universal eldercare system, where services are heavily subsidized and available solely on the basis of assessment of the individual’s support needs [10], the presence of a disability per se does not always imply that the individual themselves experiences a need to apply for support [11, 12]. A few European studies have shown that most people are already frail when entering eldercare [13], that their support needs have often become extensive before first-time use [14, 15] and that older age and a higher prevalence of chronic conditions are examples of factors that make people more likely to enter eldercare for the first time [16]. In Sweden, most people who already use eldercare (prevalence as opposed to incidence of use) belong to the oldest age groups, are of female sex, live alone, and experience a high complexity of multimorbidity [17]. However, knowledge about functional ability and health status when entering eldercare is sparse. A recent study shows that, following a downward trend since 2015, one in three first-time users (29%) now enter the program with a support need covering at least four out of five personal activities of daily living (P-ADL: bathing, dressing, toileting, indoor transferring, and eating) [18].

Exploring older adults’ health status and use of healthcare prior to the incidence of eldercare use is thus essential to obtain a broad descriptive picture of their situation at this important milestone for the provision of adequate health- and social care during aging. Such knowledge can facilitate development of proactive solutions to support older adults at an early stage of functional decline and may be valuable for both primary care and eldercare providers in their mission to coordinate resources and knowledge within high-quality local care for older people.

### Aim

The aim of this study was to identify and profile older people before their first application for publicly funded eldercare services in Stockholm in terms of socio-demographic characteristics, functional status and health status, and to describe patterns of service utilization leading up to first-time use of eldercare.

## Methods

### Data and setting

**Fig. 2:**
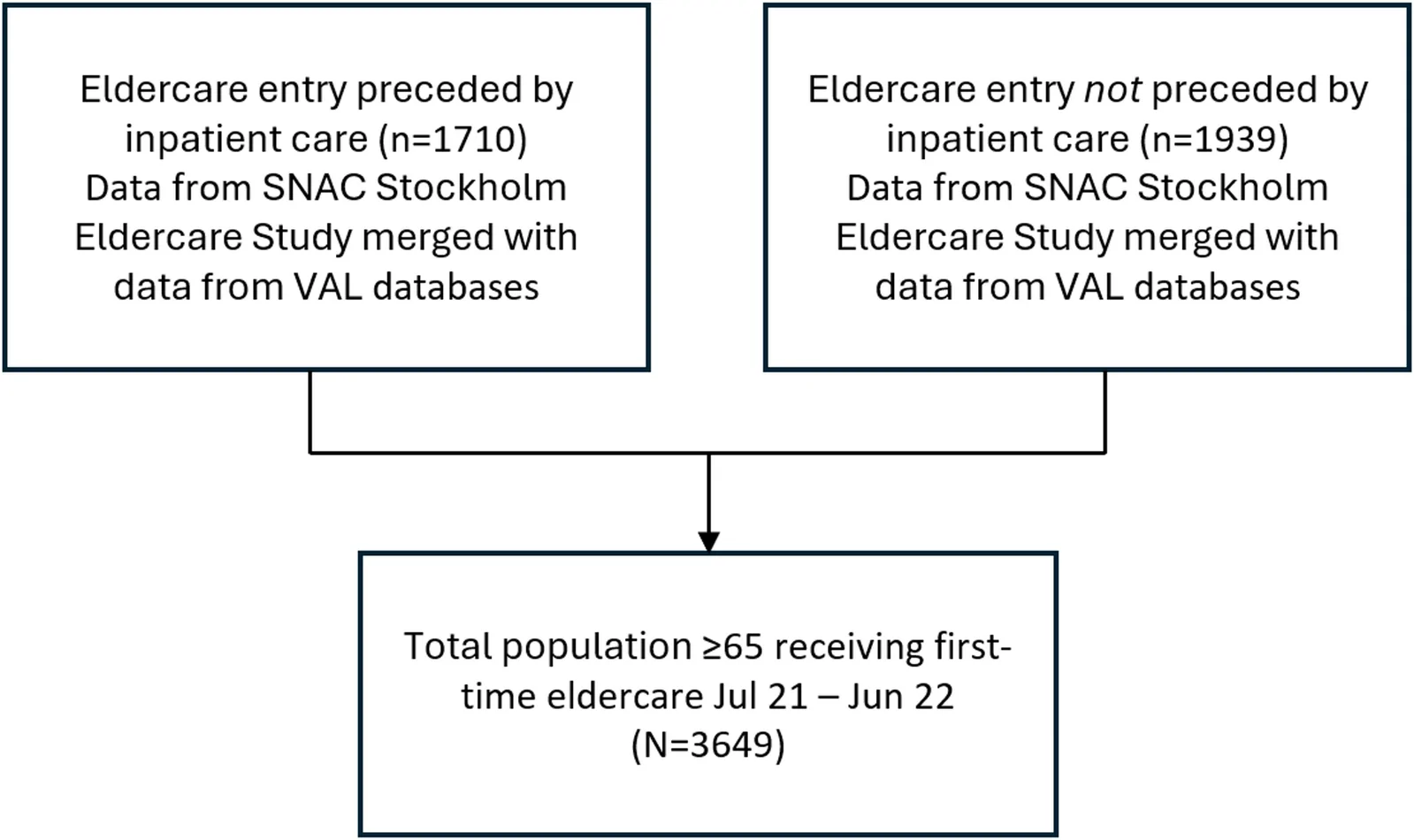
Study flowchart

Stockholm Municipality, with approximately one million inhabitants [19], is the capital of Sweden and one of 26 municipalities in Region Stockholm, covering about 10% of the Swedish population. Inhabitants aged 65 years and over constituted 15.8% of the municipal population in 2022 [20]. In this study, eldercare was coded as home care and residential care. Although other services, such as day care, escort services, and short-term stays in residential care facilities, are likewise provided following a municipal needs assessment in Sweden, home care and residential care are the only service types provided to an extent that permits meaningful statistical analysis. The study comprised all individuals ≥ 65 years who entered the eldercare program receiving either home care or residential care for the first time between July 2021 and June 2022 in the Stockholm Municipality (data retrieved in January 2023; *n* = 3649, Fig. 2).

### Data access and cleaning

Data on functional status, amount and type of eldercare, demographic variables and frequency of informal care come from the Swedish National Study on Aging and Care (SNAC) Stockholm Eldercare study [21] which includes sociodemographic information as well as data from needs assessments conducted by municipal assessors during the eldercare application process. These assessments provide a comprehensive evaluation of an individual’s life situation, including activities of daily living, social network resources, and socio-emotional well-being. The municipal assessment and documentation process is structured and standardized [22, 23]. The documentation system covers all eldercare grant decisions in the Municipality of Stockholm. Data are retrieved annually and subjected to systematic quality checks, cleaning, and classification for research purposes. These procedures are performed by data managers at the Stockholm Gerontology Research Center using structured and standardized routines to ensure high data quality and accurate representation of the original records, including preparation for linkage with other datasets.

Data on medical diagnoses, outpatient and inpatient care use were retrieved from the Region Stockholm’s storage of administrative healthcare data for analyses and follow up, the so-called VAL database. This database continuously collects administrative data on all publicly financed healthcare provided by Region Stockholm [24]. As private medical insurance is strictly regulated and accounted for 0.7% of total healthcare costs in 2019, VAL covers almost all healthcare provided [25]. For more information about the VAL database, see Carlsson et al. [26].

### Variables

This study and related analysis (see statistical analysis section below) were descriptive in focus and content. In the following subsections, the codes that were used to classify the variables are described. Future analyses will consider, for example, multivariate regression including confounders and moderating variables.

### Demographics

Age was used both as a continuous and a categorical measure (divided into two groups: 65–79 and 80 + years).

Sex was register-recorded and coded as male or female.

Living arrangements were indicated as living alone or cohabiting. Missing information for this variable was 2.2%.

### Functional status and health

In the Swedish eldercare system, home care may include assistance with limitations in instrumentational activities of daily living (I-ADL: grocery shopping, housekeeping, laundry, and meal preparation) and/or personal activities of daily living (P-ADL: bathing, dressing, toileting, indoor transferring, and eating). Data documented during the grant application process includes only P-ADL. Individuals who have been granted support, but who lack documented needs for P-ADL support can therefore be assumed to have demonstrated only a need for I-ADL support during the assessment interview. The assessment is made using Katz index of independence with six categories from 0 to 5 [27]. To ensure reasonable group sizes in this study, we merged categories 2 and 3, and 4 and 5, respectively, as these are also groups with relatively similar care needs. Missing information for this variable was 8.6%.

Cognitive problems were assessed using a shortened version of the Berger scale [28] and scored as no memory problems (Berger 0); some memory problems/being sometimes disorientated and confused (Berger 1); considerable memory problems, obvious problem, being often disorientated and confused (Berger 2–4); or severe memory problems, completely forgetful, suffering from constant disorientation and confusion (Berger 5–6). Missing information for this variable was 9.7%.

Health was assessed according to the definition of multimorbidity by Calderón-Larrañaga et al. [29] and was based on diagnoses registered in primary care, specialist care, and inpatient care during the three years preceding the onset of eldercare. The definition comprises 60 chronic disease categories derived from 918 International Classification of Diseases (ICD)-10 codes (one to four character-level). We used it to rank the most common chronic disease categories and to describe complexity of multimorbidity, categorized as 0, 1, 2, 3, 4, 5, 6, and ≥ 7 disease categories.

To describe hospital admission-related morbidity, we identified the 10 most common three-character disease categories according to ICD-10 among registered primary diagnoses at admission in those whose debut in eldercare was preceded by hospitalization. Potentially avoidable admissions were identified using the Ambulatory care-sensitive condition (ACSC) list [30], which comprises 19 conditions identified by the United Kingdom’s National Health Service. They are described as chronic, acute and preventable conditions which, from a strict medical perspective, should be possible to manage at the primary care level, provided that the healthcare system as a whole functions well. The ACSCs were identified among diagnoses recorded at the time of hospital admission.

### Informal care

The use of informal care was assessed in response to the question “Do you receive support, services or personal care from another person (partner, child/child’s partner or significant other)?” and scored as never, seldom, ≥ 1 per week, or daily. Missing information for this variable was 18.7%.

### Type of eldercare

The main forms of eldercare are home care (formal social care provided in ordinary housing) and residential care (institutional placement providing both social care and healthcare). In this study, these are the forms we refer to as type of eldercare. Amount of home care was measured as hours/month (continuous) ranging from a minimum of 0.5 h to a maximum of 152 h.

### Type of healthcare

Inpatient care was defined as any inpatient hospitalization episode coinciding with the date of being granted eldercare or during the eight days prior to the grant decision. Outpatient care was defined as primary-, specialist-, emergency- and advanced home healthcare use within six months prior to debut.

Primary care was defined as care provided at primary care centers, local emergency units, mobile emergency medical teams, or primary rehabilitation units.

Specialist care was defined as visits to any publicly subsidized outpatient specialist care clinic. In Sweden, this includes all different types of medical care (except for travel vaccines). Access almost exclusively requires medical referral, with primary care usually having the gatekeeper function.

Emergency care was defined as visits to emergency care clinics, including emergency psychiatric care clinics and emergency addiction care clinics.

Advanced home healthcare (within specialized care) was defined as home visits made by an advanced home healthcare team with competencies to meet both physical, psychological, social and existential needs. To access advanced home healthcare, a medical referral is required. Eligible patients must fulfil the criteria of needing treatment or symptom monitoring that require round-the-clock medical and nursing assessments but not being in need of inpatient care [31].

### Statistical analysis

Data from the SNAC Stockholm Eldercare Study were linked at the individual level to data from the VAL database using a pseudonymized linkage key derived from the unique personal identity numbers assigned to all residents of Sweden. Data were analyzed with descriptive statistics, using multidimensional chi-square tests for categorical data and t-tests for independent samples of continuous variables (Mann-Whitney U-tests were used as a non-parametric alternative for variables that were not normally distributed). Analyses describe the demographics, functional status, health status, informal care, and healthcare and eldercare use of all individuals. Additional descriptive analyses were also conducted with stratification according to whether the introduction of eldercare was immediately preceded by inpatient care or not. Differences between strata with a *p*-value < 0.05 were considered statistically significant. The analyses were performed with STATA 18.

### Large language models (LLMs) and reporting guidelines use

No LLMs were used in elaborating this study. The free version of DeepL Web Translator and Microsoft Copilot were used to refine the text in English. The RECORD checklist for observational studies using routinely collected health data was used as a guide in the reporting process (Additional file 1).

## Results

This study included data from all individuals ≥ 65 years who entered the eldercare program for the first time between July 2021 and June 2022 in the Stockholm Municipality (*n* = 3649) and of which 3,436 individuals were granted home care and 213 individuals were granted residential care.

Table 1 shows demographic characteristics, functional status and health indicators. The average age was 80 years. There were slightly more women than men and most individuals lived alone. Almost one in ten (8.5%) entered eldercare with considerable or severe cognitive problems and every fourth person (25.9%) had extensive need for support with P-ADL. According to the multimorbidity definition by Calderón-Larrañaga et al. [29], the vast majority (92.4%) had two or more chronic conditions and almost half (47.3%) had as many as seven or more.

**Table 1:** Demographics, functional status and health status among people receiving first-time eldercare*

Factors	All (*N* = 3649)	
	%	*N*
**Demographics**		
*Sex*		
Male	40.8	1489
Female	59.2	2160
*Age*		
Mean/median (SD)	80.7/80.9 (8.1)	
65 to 79	46.1	1679
80+	54.0	1970
*Living arrangements*		
Living alone	68.8	2458
Cohabiting	31.2	1114
**Functional status and health status**		
*Cognitive problems*		
None	65.7	2157
Some	25.8	849
Considerable	7.5	246
Severe	1.0	33
*Limitations in P-ADL*,* categories*		
None	44.9	1499
1	13.2	441
2 to 3	16.1	536
4 to 5 (extensive)	25.9	864
*Complexity of multimorbidity*, categories**		
0	4.6	169
1	3.0	111
2	4.5	165
3	7.9	287
4	10.3	374
5	11.7	426
6	11.0	401
≥ 7	47.3	1716
*Multimorbidity*, any level of complexity**		
≥ 2 categories	92.3	3369

* Data retrieved from the SNAC Stockholm Eldercare study and the VAL databases

** According to definition by Calderón−Larrañaga et al., 2016

Various cardiovascular diseases were by far the most common chronic conditions when entering eldercare. As shown in Table 2, more than half had been diagnosed with hypertension, for example. However, atrial fibrillation, heart failure, cerebrovascular diseases, and ischemic heart disease were also among the 20 most common conditions. Solid neoplasms (cancer, 24.5%), sensory impairments of vision (lens diseases, 31.9%), and hearing (29.5%), and degenerative joint diseases (24.0%) affected the life and health of one in three to four people when they entered eldercare for the first time. Roughly, diabetes was diagnosed in two out of then people, as were different intestinal disorders (colitis) and sleeping problems. Psychiatric and behavioral diseases were prevalent in 15.0% of cases. Back problems (dorsopathies), depressive state, problems involving the urinary system and kidney diseases were almost as common. Dementia, Parkinsons disease, and multiple sclerosis, all of which can have a significant impact on everyday functioning, were present in the population but were not among the 20 most common chronic diseases in the study population (Additional file 2).

**Table 2:** The 20 most common chronic conditions* among people receiving first-time eldercare**

Disease categories	All (*N* = 3649)	
	%	*N*
Hypertension	66.3	2420
Cataract and other lens diseases	31.9	1164
Deafness, hearing impairment	29.5	1078
Solid neoplasms	24.5	892
Osteoarthritis and other degenerative joint diseases	24.0	875
Atrial fibrillation	23.0	840
Diabetes	21.7	793
Colitis and related diseases	20.2	736
Other eye diseases	18.3	666
Sleep disorders	17.3	630
Anemia	17.2	628
Dyslipidemia	17.2	626
Heart failure	16.0	582
Other psychiatric and behavioral diseases	15.0	549
Cerebrovascular diseases	14.9	543
Dorsopathies	14.8	541
Ischemic heart disease	14.4	524
Depression and mood diseases	14.1	516
Other genitourinary diseases	13.8	502
Chronic kidney diseases	13.7	499

* According to definition by Calderón−Larrañaga et al., 2016

** Data retrieved from the SNAC Stockholm Eldercare study and the VAL databases

### Healthcare, informal care and eldercare characteristics at first-time eldercare use

The vast majority in this study (90.6%) had used either primary-, emergency-, specialist- or advanced home healthcare in the six months prior to their first eldercare grant decision. Although primary care was by far the most common kind of outpatient care, two in ten people did not visit primary care at all. More than half (58.4%) stated that they received informal care on a weekly or daily basis. The most common form of first-time eldercare was home care. Among individuals who received home care, those receiving the most support (fourth quartile) received an average of 110.5 h per month, while those receiving the least support (first quartile) received an average of 5.5 h per month. In other words, the extent of support granted upon entry into eldercare varies considerably, with differences of up to twenty times between people who need the most support and those who need the least (Table 3).

**Table 3:** Use of care and support among people receiving first-time eldercare*

Factors	All (*N* = 3649)	
	%	*N*
**Inpatient care**		
Immediately preceding eldercare entry	46.9	1710
**Outpatient care visits**		
six months before grant decision		
None	9.4	342
≥ 1 primary care	80.0	2919
≥ 1 specialist care	39.4	1438
≥ 1 emergency care	8.7	318
≥ 1 adv home healthcare	5.6	203
**Informal care**		
at need assessment		
Never	17.5	518
Rarely	24.1	715
≥ 1 per week	30.7	911
Daily	27.7	822
**Eldercare**		
Residiential care grant	5.8	213
Home care grant	94.2	3436
**Amount of first-time home care**		
*Hours per month*,* quartiles*		
First (least)	5.5	925
Second	15.0	832
Third	40.0	828
Fourth (most)	110.5	855

* Data retrieved from the VAL database and the SNAC Stockholm Eldercare study

Almost half of the total study population entered eldercare immediately after an episode of inpatient care, and 42.1% of those who entered eldercare after inpatient care had extensive needs for support with P-ADL (compared to 11.1% among people who entered eldercare without having received inpatient care). People who entered home care after an episode of inpatient care were granted more hours (median 31.5 h/month) than those who did not (7.5 h/month) (Table 4).

**Table 4:** Functional status and eldercare use, with stratification by previous inpatient care*

Factors	All (*N* = 3649)				*P*-value**
	Preceded by inpatient care		Not preceded by inpatient care		
	%	(*n* = 1710)	%	(*n* = 1939)	
**Functional status**					
*Limitations in P-ADL*,* categories*					
None	24.4	389	63.5	1110	< 0.000
1	12.7	203	13.6	238	
2 to 3	20.7	330	11.8	206	
4 to 5 (extensive)	42.1	671	11.1	193	
**Duration of eldercare use**					
*Three months from grant decision*					
Still receiving eldercare	67.4	1152	80.4	1558	< 0.000
No eldercare	26.1	447	16.6	321	
Deceased	6.5	111	3.1	60	
*Six months from grant decision*					
Still receiving eldercare	55.0	941	72.6	1407	< 0.000
No eldercare	35.6	609	22.3	433	
Deceased	9.4	160	5.1	99	
**Amount of first-time home care**					
Hours per month, median	31.5	1652	7.5	1775	< 0.000

* Data retrieved from the SNAC Stockholm Eldercare study and the VAL databases

** P−values refer to the significance level of differences between people whose entry into eldercare was preceded by inpatient care and those whose entry was not

There were also differences between the groups in terms of the duration of eldercare services. It was more common to continue to receive services over longer periods among those who entered eldercare without receiving inpatient care: 80.4% were still using the services after three months and 72.6% after six months. Although long-term use was less common among those who entered eldercare after an episode of inpatient care, more than half still used the services after three months (67.4%) and after six months (55.0%). The mortality rate during the six-month period after entering eldercare varied between those who entered after hospitalization (9.4%) and those whose eldercare was not preceded by hospitalization (5.1%) (Table 4).

The most common hospitalization causes and the proportion of ambulatory care sensitive conditions among individuals who entered eldercare following inpatient care are shown in Table 5 and are organized according to the ICD-10 chapters. For each chapter, we present the three most common diagnoses. As shown in the table, older adults who entered eldercare following hospitalization most often received care for severe injuries. These injuries had either external causes, with consequences such as fractures of extremities (S00-T98), or internal causes, leading to, e.g., cerebral infarction (I00-I99). However, many individuals also had been hospitalized due to their chronic ailments or related treatments, such as various types of cancer (C00-D48), declining function of the heart, blood vessels or respiratory organs (I00-I99 and J00-J99), worn condition of the musculoskeletal system (M00-M99) and impaired excretory function (N00-N99). Infections in, e.g., the air ways (J00-J99), urinary tract (N00-N99) or tissue damage/open skin areas (A00-B99) also occurred to some extent, as did various types of dizziness or impaired state of consciousness (R00-R99). Acute states of confusion without the influence of drugs or medications (F00-F99) and digestive disorders (K00-K93), mainly involving intestinal peristalsis, were also present. Of all hospitalizations that led to first-time eldercare use, 12.6% were due to Ambulatory care sensitive conditions (ACSC).

**Table 5:** The 10 most common hospitalization causes* and proportion of ambulatory care sensitive conditions (ACSC)** among people with first-time eldercare use preceded by inpatient care***

ICD 10-code group	Preceded by inpatient care	
	(*n* = 1710)	
	%	*n*
**S00-T98 - Injury**,** poisoning and certain other consequences of external causes**	22.7	387
*Most common diagnoses in this chapter*		
S72 – Fracture of femur (*n* = 141)		
S82 – Fracture of lower leg, including ankle (*n* = 47)		
S42 – Fracture of shoulder and upper arm (*n* = 35)		
**I00-I99 - Diseases of the circulatory system**	16.8	287
*Most common diagnoses in this chapter*		
I63 – Cerebral infarction (*n* = 102)		
I50 – Heart failure (*n* = 54)		
I48 – Atrial fibrillation and flutter (*n* = 18)		
**C00-D48 - Neoplasms**	9.9	168
*Most common diagnoses in this chapter*		
C34 – Malignant neoplasm of bronchus and lung (*n* = 20)		
C18 – Malignant neoplasm of colon (*n* = 15)		
C79 – Secondary malignant neoplasm of other and unspecified sites (*n* = 15)		
**M00-M99 - Diseases of the musculoskeletal system and connective tissue**	9.6	163
*Most common diagnoses in this chapter*		
M16 – Coxarthrosis [arthrosis of hip] (*n* = 30)		
M54 – Dorsalgia (*n* = 21)		
M80 – Osteoporosis with pathological fracture (*n* = 18)		
**R00-R99 - Symtoms**,** signs and abnormal clinical and laboratory findings**,** not elsewhere classified**	5.7	96
*Most common diagnoses in this chapter*		
R55 – Syncope and collapse (*n* = 22)		
R41 – Other symptoms and signs involving cognitive functions and awareness (*n* = 21)		
R42 – Dizziness and giddiness (*n* = 6)		
**J00-J99 - Diseases of the respiratory system**	5.3	91
*Most common diagnoses in this chapter*		
J44 – Other chronic obstructive pulmonary disease (*n* = 17)		
J15 – Bacterial pneumonia, not elsewhere classified (*n* = 16)		
J18 – Pneumonia, organism unspecified (*n* = 12)		
**F00-F99 - Mental**,** behavioral and neurodevelopmental disorders**	5.1	89
*Most common diagnoses in this chapter*		
F05 – Delirium, not induced by alcohol and other psychoactive substances (*n* = 22)		
F06 – Other mental disorders due to brain damage and dysfunction and to physical disease (*n* = 20)		
F00 – Dementia in Alzheimer disease (*n* = 7)		
**K00-K93 - Diseases of the digestive system**	5.1	86
*Most common diagnoses in this chapter*		
K56 – Paralytic ileus and intestinal obstruction without hernia (*n* = 14)		
K59 – Other functional intestinal disorders (*n* = 9)		
K92 – Other diseases of digestive system (*n* = 8)		
**N00-N99 - Diseases of the genitourinary system**	4.7	83
*Most common diagnoses in this chapter*		
N39 – Other disorders of urinary system (*n* = 35)		
N17 – Acute renal failure (*n* = 19)		
N10 – Acute tubulo-interstitial nephritis (*n* = 11)		
**A00-B99 - Certain infectious and parasitic diseases**	3.1	53
*Most common diagnoses in this chapter*		
A41 – Other sepsis (*n* = 15)		
A49 – Bacterial infection of unspecified site (*n* = 9)		
A46 – Erysipelas (*n* = 8)		
**Hospital admissions where ACSCs were identified**	12.6	216

* Primary diagnosis

** According to Purdy et al., 2009

*** Data retrieved from the SNAC Stockholm Eldercare study and the VAL databases

## Discussion

The results of this population-based, record-linkage study comprised all older people who entered eldercare in the largest municipality of Sweden between July 2021 and June 2022. The findings present a detailed profile of the care needs and service receipt and improve understanding about the role of socio-demographic characteristics, functional status, health status, and patterns of healthcare use. Generally, the older population entering eldercare for the first time had high levels of multimorbidity, many had extensive needs for assistance with activities of daily living, and the prevalence of psychiatric comorbidity was notable. Overall, older people were engaged with multiple forms of care, mainly including informal care, inpatient care, primary care, and home care. This example from Stockholm highlights which needs and care contexts during the period preceding the first use of publicly funded eldercare services are particularly important to consider when developing timely and well-coordinated support.

### Demographics, functional and health characteristics of individuals granted first-time eldercare

In correspondence with the few existing international studies on first-time use of eldercare [16, 32], our results show that users were mostly females and lived alone. This pattern is also consistent with Swedish eldercare use more broadly [17]. Furthermore, our findings, that just over a quarter of the population entered eldercare with extensive support needs, aligns with previous Swedish studies [18, 33].

In the Netherlands, the number of chronic conditions has been positively associated with first-time use of eldercare [16] as has the prevalence of dementia, a history of falls, depression and incontinence in Australia [32]. Direct comparisons are hard to make between eldercare systems and the morbidity categories used in these studies are not fully comparable with those applied in our analysis [32]. However, we conclude that multimorbidity appears to be a common characteristic among individuals receiving eldercare for the first time and that dementia and depression, although prevalent among people entering eldercare in Sweden, do not dominate the morbidity profile.

Compared with the results of a Swedish study that used the same multimorbidity definition [29] but examined a slightly younger sample of the general population (aged 60 + years), the complexity of multimorbidity was significantly higher in our population. While 55.8% of participants in the study by Calderón Larrañaga et al. [29], had four or more chronic conditions, as many as 79.9% in our population met this level of complexity. There were also notable differences in the composition of chronic disease clusters between the two studies. The prevalence of diseases leading to vision and hearing impairments, as well as cancer and diabetes, were significantly higher in our population. Additionally, the proportion of individuals with psychiatric and behavioral disorders was markedly higher, 15% in our study compared with 2.0% in the study by Calderón Larrañaga et al. [29].

Although differences in morbidity compared with the sample studied by Calderón Larrañaga et al. [29] are to be expected, given that our population, per definition, required support with activities in daily living, our findings regarding patterns of chronic morbidity among people with first-time use of eldercare are of substantial clinical relevance. These findings highlight needs that emerge at the onset of eldercare and can support a better understanding of the resources required as well as of how eldercare and healthcare services can optimally mobilize to meet them. Importantly, the fact that psychiatric comorbidity is present in 15.0% of individuals entering eldercare should increase awareness of the clinical and organizational complexity encountered by staff across all levels of eldercare. Psychiatric comorbidity is a topic increasingly recognized in Swedish research, where the working conditions of eldercare staff is often described as challenging [34]. With limited knowledge about the biomedical characteristics of various psychiatric conditions as well as the specific treatment and care needs of those affected, staff report frustration and feelings of inadequacy [34].

### The relationship between the use of informal and formal care

Among older adults in Sweden who already receive eldercare services, informal care accounts for by far the largest proportion of the total support provided to meet their care needs [35]. Interestingly, our findings provide a snapshot of informal care use at entry into publicly funded eldercare services, indicating that more than half of individuals received informal care at least weekly, and nearly one-third received such support daily. This occurred at a time when 80% had recently made one or more visits to primary care, 39% had visited specialist care, 9% had visited the emergency department, and 47% were hospitalized at the time of their first eldercare service grant. These findings confirm that, for many older adults, entry into the eldercare system marks a period of escalating care and support needs, during which several formal and informal care providers become involved. These results also raise questions about how older people’s support needs affect their family members and friends, and how these close relationships are subsequently affected during the period leading up to the first eldercare decision. This has recently been problematized in another study on first-time eldercare use [36].

### Functional and health-related characteristics when eldercare is preceded by inpatient care

Entering eldercare with extensive care needs may be due to a sudden deterioration in health and functioning, which is also indicated by almost half of the study population entering eldercare immediately following inpatient care. Having analyzed the causes of hospitalization (Table 4), we can confirm that the primary diagnoses reported in many cases referred to some kind of injury, often fractures of extremities. Femur, wrist and humerus fractures are a classic pattern among fall-related injuries, particularly among older adults who have reduced flexibility, strength, reaction time and balance [37]. Among first-time users of eldercare in Australia, fractures, a history of falls, incontinence, delirium and dementia - which are all diagnoses associated with falls or the risk of falls - were found to be among the most common [32]. Older people are also particularly at risk of falling due to age-related difficulty in building and maintaining muscle mass and strength [38] and impaired sensory functions, primarily visual decline [39]. The use of certain medications common in geriatric populations with chronic multimorbidity also contributes to the high prevalence of other risk factors, such as hypotension, visual impairment, somnolence, confusion, movement disorders, and dizziness [40].

Although an abrupt decline in functional ability often appears to trigger first-time eldercare use when preceded by inpatient care, it is likely that the gradual development of an underlying risk profile, characterized by complex combinations of chronic diseases and their treatments, as seen in Table 2, is the fundamental driver of injuries resulting from accidents and falls. The high prevalence of cardiovascular diseases at the time of entry into eldercare, observed both in our and other studies [32], illustrates this. It implies that the need for preventive, risk-reducing measures and support arises long before the time at which extensive functional loss becomes evident, e.g. after a fall.

Our analyses also show that the majority of those who enter eldercare following inpatient care continued to use the services after three and even six months. This suggests that many have fundamental and ongoing needs for support that cannot easily be met through conventional rehabilitation measures or recovery from the initial injuries alone. It is likely that some individuals will not regain full function after such a substantial deterioration, because their organ systems were already compromised prior to the incident. Others may have experienced impaired functioning long before receiving eldercare triggered by an acute event such as a fall.

In addition to injuries, we can conclude that many who begin receiving eldercare after an inpatient care episode have received this care due to deterioration or complications of existing chronic diseases or to temporary infections. At the same time, in a certain proportion of these hospital admissions (12.6%), ambulatory care sensitive conditions [41] were identified, suggesting that they could have been managed at the primary care level. Our analysis also shows that most people visit primary care shortly before they enter eldercare for the first time, which means that they are known to the local healthcare network. This is an opportunity for collaboration and development of high-quality local care for older people, in line with the coordinating assignment that primary care according to law and guidelines is appointed to lead [6–8].

Because of complicating comorbidities and the fact that primary care services are not available around the clock, it is still not possible to determine whether individuals who entered eldercare after inpatient treatment for ambulatory care sensitive conditions could, in fact, have been managed within primary care instead. Recovery from illness at an older age and under the influence of multiple chronic underlying diseases is always a matter that encompasses various possible scenarios. It is not possible, and less during ageing than otherwise, to regard a health incident as an isolated phenomenon. It is, however, known, that inpatient care can affect many aspects of life and health negatively for older people [42, 43], further complicating their care pathway and increasing total costs. This means that, regardless of how we define the possibility of treating specific diagnoses at different levels of care, it is important to optimize measures for healthy aging and the monitoring of chronic conditions within primary care.

Our results underscore the need for resources in primary care to work structurally and methodically with fall prevention and accident risk reduction, as well as the mitigation of underlying disease exacerbations and the rapid detection and treatment of temporary, local infections. We also suggest that there may be potential for earlier identification of support needs among older people, particularly regarding support for increased knowledge and motivation for fall preventive measures. Using the tools and spirit proposed in existing guidelines and laws on high-quality local care of older people [6–8], the expertise and resources that currently operate in a partially disconnected manner, both within municipal eldercare and within primary care, could be utilized even more effectively. Through increased cooperation and joint planning of services aimed at people not yet introduced to the eldercare program, and involving significant others of those individuals who have access to a social network, there is still potential to develop how we think of, mobilize, and implement risk-preventive care and support at the onset of functional decline due to multimorbidity in old age [44]. With reference to previous research in Sweden, showing an increase in recent years of people entering eldercare with extensive support needs [18], we also see a need to follow up on the development of multimorbidity complexity, the profile of chronic and other conditions and the use of healthcare over time. This can support the prioritization of preventive measures and facilitate the preparation of eldercare and healthcare for future needs.

### Strengths and limitations

While national register data on the use of eldercare only provides individual-based data, the SNAC Stockholm Eldercare study also provides rarely available information on an individual’s broader life circumstances regarding functional limitations and P-ADL disabilities, and informal care.

A limitation is that our data from grant assessments are restricted to the municipality of Stockholm. There is therefore a possibility that a few individuals in our data appear as first-time eldercare users, even though they may have previously used eldercare services in another municipality. However, if so, we have no reason to believe that users moving in from other municipalities would reach a number that would noteworthy affect our results. Another limitation is that the proportion of missing values in the variable for the use of informal care is high (18.7%). The response options (never, rarely, ≥ 1 per week, or daily) may also be difficult to interpret as they do not specify exact quantities. Although this variable provides vague results, we chose to include it since informal care is strongly linked to older people’s healthcare and eldercare use. It should also be mentioned as a limitation of the study that we lack data on the use of privately purchased household services, which, particularly in the form of tax-deductible cleaning services, have increased in prevalence among older adults in Sweden in recent years [45].

### Conclusions and policy implications

Our findings show that most individuals entering eldercare for the first time experienced complex, chronic multimorbidity. We highlight that a quarter had substantial functional disabilities and 15.0% had chronic, psychiatric comorbidity - parameters that significantly increase the complexity of support needs. The findings further indicate that older people were engaged with multiple forms of care, mainly including informal care, inpatient care, primary care, and, following the introduction of eldercare, also home care. The results shed light on which needs and care contexts during the period preceding the first use of publicly funded eldercare services are particularly important to consider when developing timely and well-coordinated support. Our results can inform and guide collaborative strategies, facilitating the use of expertise and methods by both municipal actors and primary care providers and their integration with the wishes and needs of older people and their significant others.

## Supplementary Information

Below is the link to the electronic supplementary material.

Supplementary Material 1: Additional file 1 Completed RECORD checklist for reporting observational studies using routinely collected health data

Supplementary Material 2: Additional Table 2 Chronic conditions among people receiving first-time eldercare (complete list)

## Data Availability

The data that support the findings of this study are available from Region Stockholm and the Stockholm Gerontology Research Center upon request. Restrictions apply to the availability of these data, which were used after authorization from the Ethics Review Authority for the current study and data are therefore not publicly available.

## Abbreviations

RECORD: Reporting of studies conducted using observational routinely-collected health data
P-ADL: Personal activities of daily living
I-ADL: Instrumental activities of daily living
SNAC: Swedish National Study on Aging and Care
ICD: International classification of diseases
ACSC: Ambulatory care-sensitive condition
LLM: Large language model
SWEAH: Swedish national graduate school on ageing and health
