# Supplementary material for: What characterizes first-time use of eldercare? A SNAC Stockholm Eldercare registry-based study of older people’s health and care needs in Sweden
Source: BMC Health Serv Res. 2026 Sep 2;26:1231. doi: 10.1186/s12913-026-15500-3 (PMC13540808; doi:10.1186/s12913-026-15500-3)
Supplement: Supplementary file 2 [file 12913_2026_15500_MOESM2_ESM.docx]

**Title**

What characterizes first-time use of eldercare? A SNAC Stockholm Eldercare registry-based study of older people’s health and care needs in Sweden.

**Journal name**

BMC health service research

**Author names, affiliations and e-mail addresses**

Pernilla Alencar Siljehag; ^1, 2^, pernilla.alencarsiljehag@aldrecentrum.se

Åsa von Berens ^1, 2^, asa.vonberens@aldrecentrum.se

Bettina Meinow ^1, 2^, bettina.meinow@aldrecentrum.se

Carin Lennartsson ^2, 3^, carin.lennartsson@ki.se

Janne Agerholm ^2^, janne.agerholm@ki.se

^1^ Stockholm Gerontology Research Center, Stockholm, Sweden

^2^ Aging Research Center, Karolinska Institutet, Stockholm University, Stockholm, Sweden

^3.^ Swedish Institute for Social Research, Stockholm University, Stockholm, Sweden

| **Additional file 2** Chronic conditions* among people receiving first-time eldercare** | | | | | | | | | | |
| --- | --- | --- | --- | --- | --- | --- | --- | --- | --- | --- |
|  |  | |  | |  | |  | |  | |
| **Disease categories** | **All (N=3649)** | | | |  | |  | |  | |
|  | % | | N | |  | |  | |  | |
| Hypertension | 66.3 | 2420 | |  | |  | |  | |  |
| Cataract and other lens diseases | 31.9 | 1164 | |  | |  | |  | |  |
| Deafness, hearing impairment | 29.5 | 1078 | |  | |  | |  | |  |
| Solid neoplasms | 24.5 | 892 | |  | |  | |  | |  |
| Osteoarthritis and other degenerative joint diseases | 24.0 | 875 | |  | |  | |  | |  |
| Artrial fibrilation | 23.0 | 840 | |  | |  | |  | |  |
| Diabetes | 21.7 | 793 | |  | |  | |  | |  |
| Colitis and related diseases | 20.2 | 736 | |  | |  | |  | |  |
| Other eye diseases | 18.3 | 666 | |  | |  | |  | |  |
| Sleep disorders | 17.3 | 630 | |  | |  | |  | |  |
| Anemia | 17.2 | 628 | |  | |  | |  | |  |
| Dyslipidemia | 17.2 | 626 | |  | |  | |  | |  |
| Heart failure | 16.0 | 582 | |  | |  | |  | |  |
| Other psychiatric and behavioral diseases | 15.0 | 549 | |  | |  | |  | |  |
| Cerebrovascular diseases | 14.9 | 543 | |  | |  | |  | |  |
| Dorsopathies | 14.8 | 541 | |  | |  | |  | |  |
| Ischemic heart disease | 14.4 | 524 | |  | |  | |  | |  |
| Depression and mood diseases | 14.1 | 516 | |  | |  | |  | |  |
| Other genitourinary diseases | 13.8 | 502 | |  | |  | |  | |  |
| Chronic kidney diseases | 13.7 | 499 | |  | |  | |  | |  |
| COPD, emphysema, chronic bronchitis | 13.5 | 493 | |  | |  | |  | |  |
| Neurotic, stress related, and somatoform diseases | 12.8 | 468 | |  | |  | |  | |  |
| Thyroid diseases | 12.7 | 465 | |  | |  | |  | |  |
| Other musculosceletar and joint diseases | 12.6 | 458 | |  | |  | |  | |  |
| Other metabolic diseases | 10.9 | 399 | |  | |  | |  | |  |
| Osteoporosis | 10.2 | 373 | |  | |  | |  | |  |
| Other cardiovascular diseases | 9.7 | 354 | |  | |  | |  | |  |
| Inflammatory arthropathies | 9.3 | 340 | |  | |  | |  | |  |
| Prostate diseases | 8.7 | 318 | |  | |  | |  | |  |
| Dementia | 8.6 | 315 | |  | |  | |  | |  |
| Autoimmune diseases | 8.3 | 302 | |  | |  | |  | |  |
| Asthma | 7.9 | 288 | |  | |  | |  | |  |
| Cardiac valve diseases | 7.8 | 284 | |  | |  | |  | |  |
| Esophagus, stomach, and duodenum diseases | 7.7 | 281 | |  | |  | |  | |  |
| Peripheral neuropathy | 7.5 | 274 | |  | |  | |  | |  |
| Glaucoma | 7.2 | 262 | |  | |  | |  | |  |
| Other neurological diseases | 6.9 | 251 | |  | |  | |  | |  |
| Venous and lymphatic diseases | 6.4 | 232 | |  | |  | |  | |  |
| Chronic ulcer of the skin | 6.2 | 227 | |  | |  | |  | |  |
| Obesity | 5.3 | 194 | |  | |  | |  | |  |
| Bradycardias and conduction diseases | 5.2 | 188 | |  | |  | |  | |  |
| Ear, nose, throat diseases | 4.4 | 160 | |  | |  | |  | |  |
| Peripheral vascular disease | 3.8 | 140 | |  | |  | |  | |  |
| Allergy | 3.4 | 125 | |  | |  | |  | |  |
| Parkinson and parkinsonism | 3.0 | 108 | |  | |  | |  | |  |
| Hematological neoplasms | 2.9 | 104 | |  | |  | |  | |  |
| Blindness, visual impairment | 2.6 | 96 | |  | |  | |  | |  |
| Chronic liver diseases | 2.3 | 84 | |  | |  | |  | |  |
| Chronic infectious diseases | 2.1 | 78 | |  | |  | |  | |  |
| Migraine and facial pain syndromes | 2.1 | 76 | |  | |  | |  | |  |
| Other respiratory diseases | 2.1 | 76 | |  | |  | |  | |  |
| Blood and blood forming organ diseases | 2.1 | 75 | |  | |  | |  | |  |
| Epilepsy | 2.0 | 72 | |  | |  | |  | |  |
| Chronic pancreas, biliary tract and gallbladder diseases | 2.0 | 71 | |  | |  | |  | |  |
| Other skin diseases | 1.7 | 62 | |  | |  | |  | |  |
| Other digestive diseases | 1.7 | 61 | |  | |  | |  | |  |
| Inflammatory bowel dieases | 1.3 | 47 | |  | |  | |  | |  |
| Schizophrenia and delusional diseases | 1.2 | 42 | |  | |  | |  | |  |
| Multiple sclerosis | 0.2 | 8 | |  | |  | |  | |  |
| ^* According to definition by Calderón-Larrañaga et al., 2016^ |  | |  | |  | |  | |  | |
| ^** Data retrieved from the SNAC Stockholm Eldercare study and VAL databases^ |  | |  | |  | |  | |  | |
